# 1-Benzyl-1*H*-benzotriazole 3-oxide–1-hy­droxy-1*H*-benzotriazole (1/1)

**DOI:** 10.1107/S1600536812030061

**Published:** 2012-07-10

**Authors:** P. Selvarathy Grace, Samuel Robinson Jebas, B. Ravindran Durai Nayagam, Dieter Schollmeyer

**Affiliations:** a Department of Chemistry, Popes College, Sawyerpuram 628 251, Tamilnadu, India; b Department of Physics, Sethupathy Government Arts College, Ramanathapuram 623 502, Tamilnadu, India; c Institut für Organische Chemie, Universität Mainz, Duesbergweg 10-14, 55099 Mainz, Germany

## Abstract

In the title compound, C_6_H_5_N_3_O·C_13_H_11_N_3_O, the benzo­triazole ring system in the 1-benzyl-1*H*-benzotriazole 3-oxide (*A*) mol­ecule is close to being planar (r.m.s. deviation = 0.011 Å); its mean plane forms a dihedral angle of 67.56 (7)° with that of the attached phenyl ring. The benzotriazole ring system in the 1-hy­droxy­benzotriazole (*B*) mol­ecule is also close to being planar (r.m.s. deviation = 0.010 Å). In the crystal, weak C—H⋯O and C—H⋯π inter­actions are present. The *A* and *B* molecules are linked by an O—H⋯N hydrogen bond.

## Related literature

For related structures and background to benzotriazoles, see: Ravindran *et al.* (2009 ▶); Selvarathy Grace *et al.* (2012 ▶).
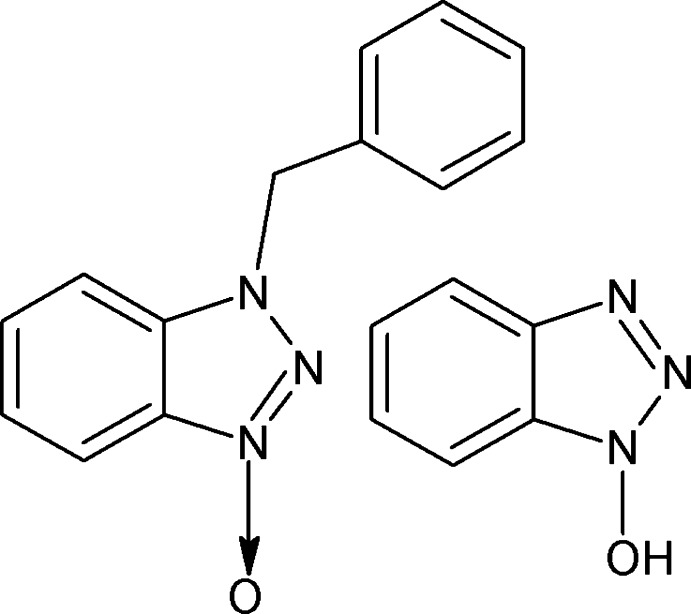


## Experimental

### 

#### Crystal data

C_6_H_5_N_3_O·C_13_H_11_N_3_O
*M* *_r_* = 360.38
Monoclinic, 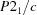

*a* = 11.2728 (8) Å
*b* = 12.2354 (5) Å
*c* = 13.1002 (9) Å
β = 110.946 (3)°
*V* = 1687.47 (18) Å^3^
*Z* = 4
Cu *K*α radiation
μ = 0.80 mm^−1^
*T* = 193 K
0.40 × 0.40 × 0.30 mm

#### Data collection

Enraf–Nonius CAD-4 diffractometer
3364 measured reflections
3197 independent reflections
2980 reflections with *I* > 2σ(*I*)
*R*_int_ = 0.102
3 standard reflections every 60 min intensity decay: 2%

#### Refinement

*R*[*F* ^2^ > 2σ(*F* ^2^)] = 0.048
*wR*(*F* ^2^) = 0.132
*S* = 1.08
3197 reflections
245 parameters
H-atom parameters constrained
Δρ_max_ = 0.35 e Å^−3^
Δρ_min_ = −0.27 e Å^−3^

### 

Data collection: *CAD-4 Software* (Enraf–Nonius, 1989 ▶); cell refinement: *CAD-4 Software*; data reduction: *CORINC* (Dräger & Gattow, 1971 ▶; Wiehl & Schollmeyer, 1994 ▶); program(s) used to solve structure: *SHELXS97* (Sheldrick, 2008 ▶); program(s) used to refine structure: *SHELXL97* (Sheldrick, 2008 ▶); molecular graphics: *SHELXTL* (Sheldrick, 2008 ▶); software used to prepare material for publication: *PLATON* (Spek, 2009 ▶).

## Supplementary Material

Crystal structure: contains datablock(s) global, I. DOI: 10.1107/S1600536812030061/hb6878sup1.cif

Structure factors: contains datablock(s) I. DOI: 10.1107/S1600536812030061/hb6878Isup2.hkl

Supplementary material file. DOI: 10.1107/S1600536812030061/hb6878Isup3.cml

Additional supplementary materials:  crystallographic information; 3D view; checkCIF report

## Figures and Tables

**Table 1:** Hydrogen-bond geometry (Å, °)

*D*—H⋯*A*	*D*—H	H⋯*A*	*D*⋯*A*	*D*—H⋯*A*
O2—H2*A*⋯N3^i^	0.84	2.57	3.3621 (18)	157
C3—H3⋯O1^ii^	0.95	2.50	3.200 (2)	130
C7—H7*B*⋯*Cg*1^iii^	0.99	2.85	3.5146 (17)	125
C18—H18⋯*Cg*1^iv^	0.95	2.69	3.510 (2)	145

Symmetry codes: (i) 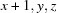; (ii) 
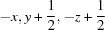
; (iii) 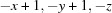; (iv) 
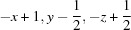
.

**Fig. 1.**
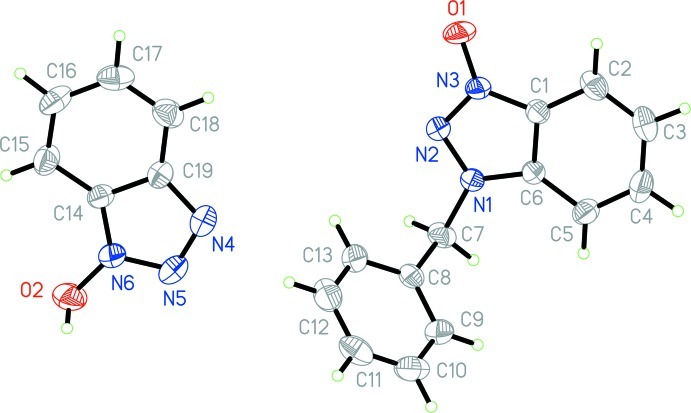
The asymmetric unit of the title compound with displacement ellipsoids drawn at the 50% probability level.

**Fig. 2.**
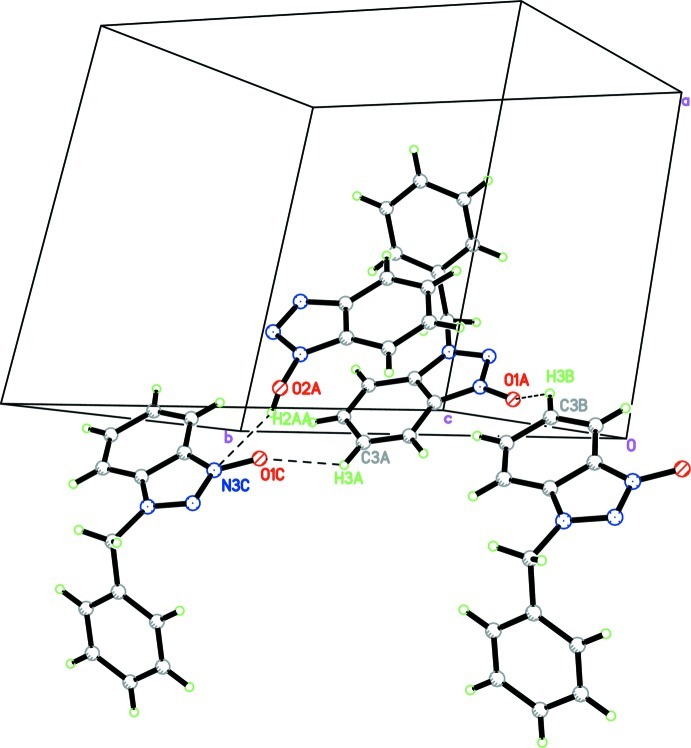
The unit cell showing the hydrogen bonding interaction of the title compound. Hydrogen bonds are shown as dashed lines.

## supplementary crystallographic information

### Comment

As part of our ongoing studies of benzotriazole derivatives (Ravindran *et al.*, 2009; Selvarathy Grace *et al.*, 2012), we now report the crystal structure of the title compound (I), (Fig. 1).

The benzotriazole rings are essentially planar with the maximum deviation from planarity being 0.015 (14) Å for atoms N1 and N5. The mean plane of the benzotriazole ring N1—N3/C1—C6 forms a dihedral angle of 67.56 (7) Å with the mean plane of the phenyl ring (C8—C13).

The crystal packing features weak C—H···π interactions. The hydrogen bonding interactions are shown in Fig 2.

### Experimental

A mixture of the sodium salt of 1- hydroxyl benzotriazole (0.314 g, 2 mmol) and benzyl chloride (0.126 g, 1 mmol) in methanol (10 ml), were heated at 333K with stirring for 6 hours. The mixture was kept aside for slow evaporation. After a week, colourless blocks were recovered.

### Refinement

H atoms were positioned geometrically [C—H = 0.95 (aromatic) or 0.99 Å (methylene)] and refined using a riding model, with *U*_iso_(H) = 1.2*U*_eq_(C).

### Crystal data

**Table:**

C_6_H_5_N_3_O·C_13_H_11_N_3_O	*F*(000) = 752
*M**_r_* = 360.38	*D*_x_ = 1.419 Mg m^−^^3^
Monoclinic, *P*2_1_/*c*	Cu *K*α radiation, λ = 1.54178 Å
Hall symbol: -P 2ybc	Cell parameters from 25 reflections
*a* = 11.2728 (8) Å	θ = 65–69°
*b* = 12.2354 (5) Å	µ = 0.80 mm^−^^1^
*c* = 13.1002 (9) Å	*T* = 193 K
β = 110.946 (3)°	Block, colourless
*V* = 1687.47 (18) Å^3^	0.40 × 0.40 × 0.30 mm
*Z* = 4	

### Data collection

**Table:**

Enraf–Nonius CAD-4 diffractometer	*R*_int_ = 0.102
Radiation source: rotating anode	θ_max_ = 70.0°, θ_min_ = 4.2°
Graphite monochromator	*h* = 0→13
ω/2θ scans	*k* = 0→14
3364 measured reflections	*l* = −15→14
3197 independent reflections	3 standard reflections every 60 min
2980 reflections with *I* > 2σ(*I*)	intensity decay: 2%

### Refinement

**Table:**

Refinement on *F*^2^	Secondary atom site location: difference Fourier map
Least-squares matrix: full	Hydrogen site location: inferred from neighbouring sites
*R*[*F*^2^ > 2σ(*F*^2^)] = 0.048	H-atom parameters constrained
*wR*(*F*^2^) = 0.132	*w* = 1/[σ^2^(*F*_o_^2^) + (0.0776*P*)^2^ + 0.6089*P*] where *P* = (*F*_o_^2^ + 2*F*_c_^2^)/3
*S* = 1.08	(Δ/σ)_max_ < 0.001
3197 reflections	Δρ_max_ = 0.35 e Å^−^^3^
245 parameters	Δρ_min_ = −0.27 e Å^−^^3^
0 restraints	Extinction correction: *SHELXL97* (Sheldrick, 2008), Fc^*^=kFc[1+0.001xFc^2^λ^3^/sin(2θ)]^-1/4^
Primary atom site location: structure-invariant direct methods	Extinction coefficient: 0.0035 (5)

### Special details

**Table:**

Geometry. All esds (except the esd in the dihedral angle between two l.s. planes) are estimated using the full covariance matrix. The cell esds are taken into account individually in the estimation of esds in distances, angles and torsion angles; correlations between esds in cell parameters are only used when they are defined by crystal symmetry. An approximate (isotropic) treatment of cell esds is used for estimating esds involving l.s. planes.
Refinement. Refinement of F^2^ against ALL reflections. The weighted R-factor wR and goodness of fit S are based on F^2^, conventional R-factors R are based on F, with F set to zero for negative F^2^. The threshold expression of F^2^ > 2sigma(F^2^) is used only for calculating R-factors(gt) etc. and is not relevant to the choice of reflections for refinement. R-factors based on F^2^ are statistically about twice as large as those based on F, and R- factors based on ALL data will be even larger.

### Fractional atomic coordinates and isotropic or equivalent isotropic displacement parameters (Å^2^)

**Table:**

	*x*	*y*	*z*	*U*_iso_*/*U*_eq_	
N1	0.23017 (11)	0.44814 (10)	0.07382 (10)	0.0233 (3)	
N2	0.21688 (11)	0.34031 (10)	0.08272 (10)	0.0261 (3)	
N3	0.12730 (11)	0.32935 (10)	0.12460 (10)	0.0256 (3)	
C1	0.08113 (13)	0.42800 (12)	0.14320 (11)	0.0231 (3)	
C2	−0.01438 (14)	0.45549 (14)	0.18361 (13)	0.0314 (4)	
H2	−0.0618	0.4017	0.2048	0.038*	
C3	−0.03466 (15)	0.56486 (15)	0.19043 (13)	0.0351 (4)	
H3	−0.0985	0.5880	0.2172	0.042*	
C4	0.03627 (15)	0.64472 (14)	0.15895 (13)	0.0340 (4)	
H4	0.0191	0.7197	0.1663	0.041*	
C5	0.12920 (14)	0.61800 (12)	0.11806 (13)	0.0284 (3)	
H5	0.1760	0.6719	0.0963	0.034*	
C6	0.15024 (13)	0.50592 (11)	0.11070 (11)	0.0216 (3)	
C7	0.32424 (14)	0.48951 (13)	0.02917 (12)	0.0285 (3)	
H7A	0.2976	0.5627	−0.0030	0.034*	
H7B	0.3269	0.4402	−0.0299	0.034*	
C8	0.45521 (13)	0.49730 (12)	0.11541 (12)	0.0250 (3)	
C9	0.50482 (15)	0.59795 (13)	0.15887 (14)	0.0327 (4)	
H9	0.4566	0.6626	0.1333	0.039*	
C10	0.62432 (17)	0.60451 (15)	0.23944 (15)	0.0384 (4)	
H10	0.6576	0.6736	0.2691	0.046*	
C11	0.69520 (15)	0.51109 (15)	0.27686 (13)	0.0368 (4)	
H11	0.7768	0.5158	0.3326	0.044*	
C12	0.64695 (16)	0.41032 (15)	0.23292 (15)	0.0379 (4)	
H12	0.6957	0.3459	0.2583	0.045*	
C13	0.52801 (15)	0.40353 (13)	0.15226 (14)	0.0323 (4)	
H13	0.4957	0.3345	0.1217	0.039*	
O1	0.09227 (11)	0.23257 (9)	0.14562 (11)	0.0383 (3)	
N4	0.66756 (14)	0.18397 (12)	0.08895 (14)	0.0428 (4)	
N5	0.75634 (14)	0.21667 (11)	0.05304 (14)	0.0417 (4)	
N6	0.81861 (13)	0.12778 (10)	0.03929 (12)	0.0312 (3)	
C14	0.77039 (14)	0.03437 (12)	0.06416 (12)	0.0252 (3)	
C15	0.79910 (16)	−0.07638 (13)	0.06063 (13)	0.0329 (4)	
H15	0.8655	−0.1009	0.0377	0.039*	
C16	0.72455 (18)	−0.14724 (13)	0.09268 (14)	0.0372 (4)	
H16	0.7400	−0.2235	0.0921	0.045*	
C17	0.62562 (17)	−0.11034 (15)	0.12655 (14)	0.0378 (4)	
H17	0.5768	−0.1625	0.1482	0.045*	
C18	0.59823 (16)	−0.00226 (15)	0.12905 (14)	0.0353 (4)	
H18	0.5313	0.0218	0.1516	0.042*	
C19	0.67306 (15)	0.07221 (13)	0.09693 (13)	0.0293 (3)	
O2	0.91290 (12)	0.13654 (10)	−0.00258 (11)	0.0419 (3)	
H2A	0.9753	0.1692	0.0424	0.063*	

### Atomic displacement parameters (Å^2^)

**Table:**

	*U*^11^	*U*^22^	*U*^33^	*U*^12^	*U*^13^	*U*^23^
N1	0.0237 (6)	0.0214 (6)	0.0286 (6)	−0.0013 (5)	0.0138 (5)	0.0007 (5)
N2	0.0253 (6)	0.0211 (6)	0.0344 (7)	−0.0006 (5)	0.0139 (5)	−0.0006 (5)
N3	0.0235 (6)	0.0201 (6)	0.0339 (7)	−0.0012 (4)	0.0112 (5)	0.0047 (5)
C1	0.0217 (6)	0.0232 (7)	0.0246 (7)	0.0012 (5)	0.0086 (5)	0.0035 (5)
C2	0.0267 (7)	0.0391 (9)	0.0321 (8)	0.0020 (6)	0.0150 (6)	0.0055 (7)
C3	0.0309 (8)	0.0452 (10)	0.0327 (8)	0.0095 (7)	0.0157 (7)	−0.0010 (7)
C4	0.0361 (8)	0.0288 (8)	0.0355 (8)	0.0080 (6)	0.0109 (7)	−0.0055 (6)
C5	0.0311 (8)	0.0215 (7)	0.0322 (8)	−0.0004 (6)	0.0108 (6)	0.0006 (6)
C6	0.0210 (6)	0.0215 (7)	0.0226 (7)	0.0002 (5)	0.0081 (5)	0.0004 (5)
C7	0.0290 (8)	0.0327 (8)	0.0294 (8)	−0.0041 (6)	0.0173 (6)	0.0016 (6)
C8	0.0264 (7)	0.0273 (7)	0.0280 (7)	−0.0023 (6)	0.0179 (6)	−0.0000 (6)
C9	0.0350 (8)	0.0258 (8)	0.0426 (9)	−0.0038 (6)	0.0203 (7)	0.0017 (6)
C10	0.0405 (9)	0.0372 (9)	0.0416 (9)	−0.0148 (7)	0.0197 (7)	−0.0068 (7)
C11	0.0292 (8)	0.0537 (11)	0.0301 (8)	−0.0065 (7)	0.0138 (6)	0.0012 (7)
C12	0.0342 (8)	0.0414 (9)	0.0415 (9)	0.0066 (7)	0.0177 (7)	0.0076 (7)
C13	0.0334 (8)	0.0287 (8)	0.0407 (9)	0.0001 (6)	0.0206 (7)	−0.0030 (6)
O1	0.0371 (6)	0.0216 (6)	0.0573 (8)	−0.0058 (4)	0.0181 (5)	0.0105 (5)
N4	0.0421 (8)	0.0264 (7)	0.0647 (10)	0.0033 (6)	0.0249 (7)	−0.0049 (7)
N5	0.0449 (8)	0.0207 (7)	0.0623 (10)	0.0006 (6)	0.0225 (7)	0.0008 (6)
N6	0.0332 (7)	0.0223 (6)	0.0432 (8)	−0.0021 (5)	0.0199 (6)	0.0021 (5)
C14	0.0301 (7)	0.0211 (7)	0.0261 (7)	−0.0034 (6)	0.0121 (6)	−0.0010 (5)
C15	0.0427 (9)	0.0258 (8)	0.0357 (8)	0.0037 (7)	0.0208 (7)	−0.0014 (6)
C16	0.0550 (11)	0.0203 (7)	0.0382 (9)	−0.0034 (7)	0.0189 (8)	−0.0006 (6)
C17	0.0452 (10)	0.0367 (9)	0.0342 (9)	−0.0141 (7)	0.0175 (7)	0.0008 (7)
C18	0.0343 (8)	0.0419 (9)	0.0350 (9)	−0.0054 (7)	0.0187 (7)	−0.0043 (7)
C19	0.0313 (8)	0.0247 (8)	0.0327 (8)	0.0001 (6)	0.0124 (6)	−0.0043 (6)
O2	0.0451 (7)	0.0395 (7)	0.0521 (7)	−0.0101 (5)	0.0309 (6)	−0.0005 (6)

### Geometric parameters (Å, º)

**Table:**

N1—N2	1.3376 (17)	C10—H10	0.9500
N1—C6	1.3625 (18)	C11—C12	1.387 (3)
N1—C7	1.4718 (17)	C11—H11	0.9500
N2—N3	1.3171 (17)	C12—C13	1.381 (2)
N3—O1	1.3082 (16)	C12—H12	0.9500
N3—C1	1.3703 (19)	C13—H13	0.9500
C1—C6	1.391 (2)	N4—N5	1.311 (2)
C1—C2	1.400 (2)	N4—C19	1.371 (2)
C2—C3	1.366 (2)	N5—N6	1.3411 (19)
C2—H2	0.9500	N6—C14	1.3545 (19)
C3—C4	1.414 (3)	N6—O2	1.3635 (17)
C3—H3	0.9500	C14—C19	1.393 (2)
C4—C5	1.376 (2)	C14—C15	1.398 (2)
C4—H4	0.9500	C15—C16	1.373 (2)
C5—C6	1.401 (2)	C15—H15	0.9500
C5—H5	0.9500	C16—C17	1.414 (3)
C7—H7A	0.9900	C16—H16	0.9500
C7—H7B	0.9900	C17—C18	1.361 (3)
C8—C9	1.388 (2)	C17—H17	0.9500
C8—C13	1.393 (2)	C18—C19	1.404 (2)
C9—H9	0.9500	C18—H18	0.9500
C10—C11	1.380 (3)	O2—H2A	0.8400
			
N2—N1—C6	111.83 (11)	C11—C10—C9	120.30 (16)
N2—N1—C7	119.53 (12)	C11—C10—H10	119.9
C6—N1—C7	128.63 (12)	C9—C10—H10	119.9
N3—N2—N1	105.26 (11)	C10—C11—C12	119.83 (16)
O1—N3—N2	120.92 (12)	C10—C11—H11	120.1
O1—N3—C1	126.69 (12)	C12—C11—H11	120.1
N2—N3—C1	112.38 (12)	C13—C12—C11	120.02 (16)
N3—C1—C6	105.04 (12)	C13—C12—H12	120.0
N3—C1—C2	132.16 (14)	C11—C12—H12	120.0
C6—C1—C2	122.80 (14)	C12—C13—C8	120.41 (15)
C3—C2—C1	115.44 (15)	C12—C13—H13	119.8
C3—C2—H2	122.3	C8—C13—H13	119.8
C1—C2—H2	122.3	N5—N4—C19	108.20 (14)
C2—C3—C4	122.17 (15)	N4—N5—N6	107.69 (13)
C2—C3—H3	118.9	N5—N6—C14	112.19 (13)
C4—C3—H3	118.9	N5—N6—O2	120.74 (13)
C5—C4—C3	122.55 (15)	C14—N6—O2	126.92 (13)
C5—C4—H4	118.7	N6—C14—C19	102.81 (13)
C3—C4—H4	118.7	N6—C14—C15	133.83 (14)
C4—C5—C6	115.51 (14)	C19—C14—C15	123.35 (14)
C4—C5—H5	122.2	C16—C15—C14	115.32 (15)
C6—C5—H5	122.2	C16—C15—H15	122.3
N1—C6—C1	105.47 (12)	C14—C15—H15	122.3
N1—C6—C5	133.00 (14)	C15—C16—C17	122.14 (15)
C1—C6—C5	121.52 (13)	C15—C16—H16	118.9
N1—C7—C8	112.06 (12)	C17—C16—H16	118.9
N1—C7—H7A	109.2	C18—C17—C16	121.94 (16)
C8—C7—H7A	109.2	C18—C17—H17	119.0
N1—C7—H7B	109.2	C16—C17—H17	119.0
C8—C7—H7B	109.2	C17—C18—C19	117.22 (15)
H7A—C7—H7B	107.9	C17—C18—H18	121.4
C9—C8—C13	119.20 (14)	C19—C18—H18	121.4
C9—C8—C7	120.43 (14)	N4—C19—C14	109.10 (14)
C13—C8—C7	120.37 (14)	N4—C19—C18	130.86 (15)
C10—C9—C8	120.23 (15)	C14—C19—C18	120.03 (15)
C10—C9—H9	119.9	N6—O2—H2A	109.5
C8—C9—H9	119.9		
			
C6—N1—N2—N3	0.52 (15)	C7—C8—C9—C10	178.88 (14)
C7—N1—N2—N3	179.96 (12)	C8—C9—C10—C11	0.2 (3)
N1—N2—N3—O1	−178.89 (12)	C9—C10—C11—C12	0.5 (3)
N1—N2—N3—C1	0.15 (16)	C10—C11—C12—C13	−0.3 (3)
O1—N3—C1—C6	178.25 (13)	C11—C12—C13—C8	−0.7 (2)
N2—N3—C1—C6	−0.73 (16)	C9—C8—C13—C12	1.5 (2)
O1—N3—C1—C2	−2.6 (3)	C7—C8—C13—C12	−178.64 (14)
N2—N3—C1—C2	178.37 (15)	C19—N4—N5—N6	−0.6 (2)
N3—C1—C2—C3	−179.72 (15)	N4—N5—N6—C14	1.1 (2)
C6—C1—C2—C3	−0.8 (2)	N4—N5—N6—O2	176.88 (14)
C1—C2—C3—C4	−0.1 (2)	N5—N6—C14—C19	−1.03 (18)
C2—C3—C4—C5	0.9 (3)	O2—N6—C14—C19	−176.51 (14)
C3—C4—C5—C6	−0.7 (2)	N5—N6—C14—C15	178.09 (17)
N2—N1—C6—C1	−0.96 (15)	O2—N6—C14—C15	2.6 (3)
C7—N1—C6—C1	179.66 (13)	N6—C14—C15—C16	−179.35 (17)
N2—N1—C6—C5	−179.96 (15)	C19—C14—C15—C16	−0.4 (2)
C7—N1—C6—C5	0.7 (3)	C14—C15—C16—C17	0.1 (2)
N3—C1—C6—N1	0.98 (15)	C15—C16—C17—C18	0.2 (3)
C2—C1—C6—N1	−178.23 (13)	C16—C17—C18—C19	−0.3 (3)
N3—C1—C6—C5	−179.87 (13)	N5—N4—C19—C14	−0.01 (19)
C2—C1—C6—C5	0.9 (2)	N5—N4—C19—C18	−178.73 (17)
C4—C5—C6—N1	178.71 (15)	N6—C14—C19—N4	0.61 (17)
C4—C5—C6—C1	−0.2 (2)	C15—C14—C19—N4	−178.63 (15)
C6—N1—C7—C8	94.88 (17)	N6—C14—C19—C18	179.50 (14)
N1—C7—C8—C9	−104.04 (16)	C15—C14—C19—C18	0.3 (2)
N1—C7—C8—C13	76.11 (17)	C17—C18—C19—N4	178.71 (17)
C13—C8—C9—C10	−1.3 (2)	C17—C18—C19—C14	0.1 (2)

### Hydrogen-bond geometry (Å, º)

**Table:**

*D*—H···*A*	*D*—H	H···*A*	*D*···*A*	*D*—H···*A*
O2—H2*A*···N3^i^	0.84	2.57	3.3621 (18)	157
C3—H3···O1^ii^	0.95	2.50	3.200 (2)	130
C7—H7*B*···*Cg*1^iii^	0.99	2.85	3.5146 (17)	125
C18—H18···*Cg*1^iv^	0.95	2.69	3.510 (2)	145

Symmetry codes: (i) *x*+1, *y*, *z*; (ii) −*x*, *y*+1/2, −*z*+1/2; (iii) −*x*+1, −*y*+1, −*z*; (iv) −*x*+1, *y*−1/2, −*z*+1/2.
